# Serine Protease Inhibitor 1 (SERPINE1) Regulates Epithelial-Mesenchymal Transition Gene Expression to Promote Migration and Invasion in Hepatocellular Carcinoma

**DOI:** 10.5812/ijpr-163908

**Published:** 2026-08-09

**Authors:** Run Bao, Zhaoxia Luo, Liao Gang, Wen Huang, Sheng Wu, Fangmin Xu

**Affiliations:** 1 Department of Gastroenterology, The Second Affiliated Hospital of Soochow University, Suzhou, Jiangsu, China; 2 Department of Gastroenterology, The First Affiliated Hospital of Naval Medical University (Shanghai Changhai Hospital), Shanghai 200433, China; 3 Department of Hepatobiliary Surgery, The Second Affiliated Hospital of Soochow University, Suzhou, Jiangsu, China

**Keywords:** Serine Protease Inhibitor 1 (SERPINE1), Migration and Invasion, Epithelial–mesenchymal Transition (EMT), Hepatocellular Carcinoma (HCC)

## Abstract

**Background:**

Hepatocellular carcinoma (HCC) is characterized by a high capacity for invasion and metastasis. Epithelial–mesenchymal transition (EMT) is a key mechanism that promotes tumor migration and invasion. However, the role of serine protease inhibitor 1 (SERPINE1) as a regulatory factor that may enhance tumor progression by directly modulating EMT-related gene expression in HCC has not been fully elucidated.

**Objectives:**

To investigate the role of SERPINE1 in regulating EMT-related gene expression and its effects on tumor invasion and migration in hepatocellular carcinoma (HCC).

**Methods:**

A comprehensive experimental approach integrating in vitro cell models, animal experiments, and clinical sample analyses was employed. qPCR, immunohistochemistry, and Western blotting were performed to determine SERPINE1 expression in HCC cell lines and tissues. SERPINE1 expression was modulated by RNA interference and overexpression, and the effects on HCC cell migration and invasion, as well as the expression of EMT markers, were evaluated.

**Results:**

SERPINE1 expression was approximately 3.2-fold and 2.8-fold higher in metastatic liver cancer cells and cancer tissues, respectively (P < 0.01). SERPINE1 expression was significantly negatively correlated with E-cadherin (r = -0.72) and positively correlated with vimentin (r = 0.65). After SERPINE1 knockdown, E-cadherin protein expression increased 2.1-fold, whereas N-cadherin and vimentin expression decreased by 68% and 55%, respectively; cell migration and invasion decreased by 62% and 57%, respectively (P < 0.01). Conversely, SERPINE1 overexpression reduced E-cadherin to 0.4-fold of the control level, whereas N-cadherin and vimentin increased 2.3-fold and 2.0-fold, respectively, and migration and invasion increased 1.9-fold and 2.1-fold, respectively. In the in vivo orthotopic lung metastasis model, SERPINE1 knockdown reduced the number of lung metastatic foci from 4.8 ± 1.1 to 1.5 ± 0.6 (P < 0.01), and the incidence of metastasis decreased from 83.3% to 33.3%. Mechanistic studies revealed that, after SERPINE1 knockdown, the p-AKT/AKT and p-ERK/ERK ratios decreased by 64% and 57%, respectively, and the EMT transcription factors Snail and Slug were downregulated; PI3K agonists partially reversed the inhibition of the EMT phenotype. These results suggest that SERPINE1 activates the PI3K/AKT and MAPK signaling pathways, upregulates EMT-related gene expression, and thereby promotes liver cancer cell migration, invasion, and lung metastasis.

**Conclusions:**

SERPINE1 facilitates the migration and invasion of HCC cells by modulating EMT-related gene expression. SERPINE1 is upregulated in liver cancer tissues and may contribute to liver cancer progression.

## 1. Background

Each year, the global number of new liver cancer cases and deaths exceeds 968,000 (1). Liver cancer prevalence is highest in East Asia and Eastern Europe, and the annual incidence among younger individuals is increasing. Most patients with liver cancer are diagnosed at an advanced stage, resulting in a poor prognosis and a low 5-year survival rate because of the lack of specific clinical markers. Currently, major treatment modalities, including surgery, radiotherapy, chemotherapy, targeted therapy, and immunotherapy, provide limited therapeutic benefit for patients with advanced liver cancer. Liver cancer is highly heterogeneous in its genetic characteristics and clinical manifestations. Histological classification alone is insufficient to appropriately stratify patients for individualized treatment and improve prognosis (2-4). SERPINE1, an inhibitor of urokinase and tissue plasminogen activator, could theoretically act as an antitumor factor by inhibiting tumor development. Nevertheless, studies have shown that SERPINE1 expression is upregulated in several malignancies, including breast, colorectal, gastric, and esophageal cancers, and is associated with poor prognosis (5-7). Studies have also reported that triple-negative breast cancer cells can produce SERPINE1, which acts on these cells in a paracrine manner to increase their migration and invasion (8-11).

Hepatocellular carcinoma ranks as the sixth most common malignant neoplasm worldwide. Its high metastatic potential is a major factor contributing to poor patient outcomes (12). Statistics indicate that more than 70% of patients with HCC have metastasis or metastatic potential at diagnosis, and the 5-year survival rate for metastatic HCC is below 10%. A comprehensive understanding of the molecular mechanisms underlying HCC metastasis is essential for developing novel treatments (13). Epithelial-mesenchymal transition is a critical process in tumor metastasis, during which epithelial cells acquire mesenchymal characteristics, thereby enhancing cellular motility and invasion and promoting local infiltration and distant metastasis in HCC. Research has shown that aberrant expression of multiple EMT-related genes is significantly associated with the clinical stage and prognosis of patients with HCC (14-16). Serine protease inhibitor 1 is an important protease inhibitor that is aberrantly expressed in various tumors; however, whether it participates in HCC metastasis by regulating EMT-related gene expression remains unclear. The specific function of SERPINE1 in HCC and its relationship with EMT regulation remain relatively understudied, particularly at the molecular level.

## 2. Objectives

This study aimed to elucidate the mechanism by which SERPINE1 promotes HCC cell migration and invasion by regulating EMT-related gene expression, thereby identifying a potential molecular target for understanding HCC metastasis and providing a theoretical basis for developing therapeutic strategies targeting the SERPINE1/EMT axis. We hypothesized that SERPINE1 upregulates EMT-related gene expression, enhances HCC cell migration and invasion, and thereby promotes tumor metastasis.

## 3. Methods

### 3.1. Tissue Specimens and Cells

#### 3.1.1. Sample Collection

A total of 100 paired HCC tissues and adjacent normal liver tissues were collected. All samples were obtained from surgical specimens of patients who underwent surgery at the Second Affiliated Hospital of Soochow University from January 2022 to December 2025. All patients had pathologically confirmed HCC, and none had received radiotherapy, targeted therapy, or immunotherapy before surgery. Patients were 35 - 75 years old, with a median age of 58.6 years; 72 were male and 28 were female. According to the UICC eighth-edition TNM staging criteria, 28 cases were stage I, 42 were stage II, 25 were stage III, and 5 were stage IV. Tumor diameter was ≤ 5 cm in 46 cases and > 5 cm in 54 cases.

After collection, the samples were immediately frozen in liquid nitrogen and transferred to a -80 °C ultra-low-temperature freezer for storage. All patients provided written informed consent, and the study was approved by the ethics committee (approval No. HKYS-2025-A0521). Strict aseptic procedures were followed during sample collection to preserve sample integrity and biological activity. The sample size was determined based on a previous pilot experiment. Preliminary analysis of 20 samples showed a significant difference in SERPINE1 expression between cancer and adjacent normal tissues (Cohen d = 1.2). Power analysis using GPower 3.1 (power = 0.8, α = 0.05, two-tailed test) indicated a minimum sample size of 86 cases. Considering potential data loss and sample-quality issues, 100 paired samples were collected to support the reliability and reproducibility of the statistical analyses.

### 3.2. Inclusion and Exclusion Criteria

#### 3.2.1. Inclusion Criteria

The inclusion criteria were as follows: 1) pathologically confirmed primary HCC meeting the 2023 WHO diagnostic criteria; 2) radical resection without preoperative radiotherapy or chemotherapy; and 3) complete clinicopathological data, including age, sex, TNM stage, and BCLC stage.

#### 3.2.2. Exclusion Criteria

The exclusion criteria were as follows: 1) other malignant tumors or severe extrahepatic diseases; 2) RNA degradation in tissue samples (OD260/280 < 1.8 or RIN < 7); and 3) postoperative survival of less than 3 months to exclude interference from nontumor factors.

#### 3.2.3. Animal Experiment Exclusion Criteria

In the animal experiment, mice were assigned using a completely randomized design (n = 6 mice per group). The following exclusion criteria were established: 1) modeling failure, defined as a transplanted tumor volume of less than 50 mm^3^ on day 7; and 2) exclusion of an entire batch when the intraoperative mortality rate exceeded 10%.

### 3.3. Main Reagents and Instruments

SERPINE1 small interfering RNA (siRNA) was purchased from Shanghai Jiman Biotechnology Co., Ltd. Phosphate-buffered saline (PBS), 0.25% trypsin solution, and penicillin-streptomycin solution (100×) were purchased from Gino Biomedical Technology Co., Ltd. The rabbit monoclonal SERPINE1 antibody (No. ab317604) was purchased from Abcam Corporation, United States, and the mouse monoclonal GAPDH antibody (No. AC002) was purchased from ABclonal Corporation, China. Transwell chambers and matrix adhesive were purchased from Corning Corporation, United States (17).

Complete coding-region sequence (CDS) (NM_000602.4_):

ATGAAGAGTCTGCTGCCGTCGCTGGTGCTGCTGCTGCTGC

CGCTGTGCTGGGCCCTGGCGGAG

AAGTCCATCACCGAGGAGTCGCCGGCCCGCGAGAACAAGA

TCAAGCAGGTGGAGGCCAACATC

GACATCGCCAACAAGGCCAAGCGCTACGTGGACATCGCCG

AGAGCCTGCGCAAGCTGGCCGAG

CGCTTCGACACCGCCGAGATCCGCGAGCTGGTGGCCGACA

TCGCCAAGCGCGCCGAGCTGCAG

SERPINE1 siRNA sequences:

si-SERPINE1:

5'→3' GCAACAUCGACAUCGCCAAdTdT

3'→5' UUGGCGAUGUCGAUGUUGCdTdT

Negative control siRNA:

5'→3' UUCUCCGAACGUGUCACGUdTdT

3'→5' ACGUGACACGUUCGGAGAAdTdT

### 3.4. Experimental Methods

#### 3.4.1. Experimental Group Design

The blank control group consisted of the parental HCC cell line (HepG2 or Huh7) without transfection or the animal model administered PBS/empty viral vector, and was used to establish the baseline state of the experimental system. The negative control (NC) group consisted of cells transfected with nontargeting control siRNA (scrambled siRNA) or animals injected with control vectors (empty-vector lentivirus) to account for nonspecific effects of transfection reagents or vectors on cellular behavior or animal phenotypes. The SERPINE1 knockdown groups (siRNA1 and siRNA2) consisted of cells transfected with two independently designed effective siRNA sequences targeting different coding regions of human SERPINE1 or animals injected with HCC cells stably expressing the corresponding shRNA.

#### 3.4.2. Bioinformatics Analysis

Differential expression of SERPINE1 across 33 cancer types was analyzed using the TIMER 2.0 online database, together with transcriptomic and related clinical data, including survival status and clinicopathological stage, from liver cancer tissues. Kyoto Encyclopedia of Genes and Genomes (KEGG) pathway enrichment analysis was performed to identify key signaling pathways associated with differentially expressed genes (DEGs), thereby characterizing their biological functions and potential regulatory mechanisms. SERPINE1 expression in liver cancer and adjacent tissues was analyzed using R packages, including limma, ggplot2, ggpubr, and survival. SERPINE1 expression across clinical stages of liver cancer was compared, and survival curves were generated.

#### 3.4.3. Cell Transfection

The human HCC cell lines HepG2 and Huh7 were obtained from the American Type Culture Collection (ATCC). Cells were cultured in high-glucose DMEM containing 10% fetal bovine serum (FBS) and 1% penicillin-streptomycin. Cells were maintained at 37 °C in 5% CO_2_ and 100% humidity. All cells were authenticated by STR typing and regularly tested for mycoplasma contamination. Cells were passaged using 0.25% trypsin when cell density reached 80% - 90%.

Twenty-four hours before transfection, cells were seeded at a density of 5 × 10^5^ cells per well in 6-well plates. When cell density reached 60% - 70%, jetPRIME® transfection reagent was used. According to the manufacturer's instructions, 2 μg of SERPINE1 overexpression plasmid or 50 pmol of siRNA (siRNA1, siRNA2, siRNA3, and negative-control CON siRNA) was mixed with the corresponding volume of jetPRIME® reagent in Opti-MEM medium and incubated at room temperature for 15 minutes before being added to the cell-culture wells. After 6 hours of transfection, the complete medium was replaced, and the cells were cultured for 24 and 48 hours for subsequent protein- and RNA-level analyses. All experiments were performed with 3 biological replicates to ensure reproducibility and reliability.

### 3.5. Western Blotting

HepG2 and Huh7 hepatoma cells were collected 48 hours after transfection. Total cellular protein was extracted on ice. Loading buffer was added at the appropriate ratio, and the mixture was boiled for 10 minutes to denature the proteins. A 20-μg protein sample was loaded into each lane of a 10% SDS-PAGE gel. Electrophoresis was performed at a constant voltage of 85 V for 30 minutes and then at 110 V for 1 hour.

### 3.6. RNA Extraction

After 24 hours of transfection, 100 pairs of clinical tissue samples and HepG2 and Huh7 hepatoma cells were collected. A reverse-transcription kit was used to generate cDNA. The qRT-PCR system contained 20 μL of SYBR Green Master Mix. In the RNA-interference experiment, specific siRNA sequences targeting SERPINE1 were designed and transfected into an HCC cell line, and a negative-control siRNA group was established to exclude nonspecific effects. Transfection efficiency was verified by fluorescence labeling and qPCR, ensuring gene-silencing efficiency greater than 70%. In the overexpression experiment, full-length SERPINE1 cDNA was cloned into a eukaryotic expression vector and introduced into cells by liposome-mediated transfection, with empty-vector transfection serving as the control. Both groups of cells were selected with G418 to obtain stable expression strains, and protein expression was confirmed by Western blotting to be significantly upregulated. These interventions were designed to clarify the regulatory mechanism of SERPINE1 on EMT-related genes, including E-cadherin, N-cadherin, and vimentin, and to verify its functional effects using Transwell migration and invasion assays, thereby supporting the specificity and reliability of the experimental results.

SERPINE1 gene sequence (18):

5'→3' GAAGCTGCTGGTGAAGATCG

3'→5' GCTAGAAGTGGTGTCGAAG

GAPDH gene sequence:

5'→3' GAAGGTGAAGGTCGGAGTC

3'→5' CTGAGGCTGGAAGGTGAAG

### 3.7. Animal Experiments

#### 3.7.1. Experimental Animals and Housing Conditions

Male NOD/SCID immunodeficient mice, aged 6 - 8 weeks and weighing 18 - 22 g, were purchased from Beijing Vitogen Life Science Experimental Animal Technology Co., Ltd. All mice were acclimated in SPF-level animal housing for 1 week. Environmental temperature was maintained at 22 ± 2 °C, relative humidity at 50% - 60%, and a 12-hour light-dark cycle was used. Mice had free access to food and water. All animals received the same husbandry throughout the experiment, and bedding was changed twice weekly.

#### 3.7.2. Ethics and Compliance

The experiment strictly followed the Guidelines for Ethical Review of Laboratory Animal Welfare and the 3R principles of replacement, reduction, and refinement. All animal experimental protocols were approved by the Animal Protection and Use Committee (approval No. HKYS-2025-A0521), and ethical registration was completed before the experiment began.

#### 3.7.3. Animal Model Construction and Grouping

Two animal models were constructed according to the experimental objectives: a subcutaneous tumor model and an orthotopic transplantation model. For the subcutaneous tumor model, 5 × 10^6^ Huh7 cells with stable SERPINE1 knockdown were injected subcutaneously into the right scapular region of each mouse. For the orthotopic transplantation model, 2 × 10^6^ cells were injected into the left liver lobe of each mouse. The control group received the corresponding number of empty-vector-transfected cells. Computer-generated random-number tables were used for group allocation to ensure comparable cage-position distribution and feeding conditions between the experimental and control groups. The subcutaneous tumor model included 8 mice per group, whereas the orthotopic transplantation model included 10 mice per group.

#### 3.7.4. Sample Size Determination and Endpoint Indicators

Based on the previous pilot experiment, the effect size was d = 1.5, α = 0.05, and power = 0.8. GPower 3.1 indicated a minimum sample size of n = 7 per group. Considering possible outliers, each group ultimately included n ≥ 8 animals. Experimental endpoints included tumor volume, weight change, and activity. If tumor diameter exceeded 20 mm, body weight decreased by more than 20%, or obvious impairment of activity occurred, humane euthanasia by CO_2_ asphyxiation was performed immediately.

#### 3.7.5. Tissue Sample Processing

After the experiment, the animals were euthanized, and tumor tissues or metastatic foci were completely removed. All tissue samples were immediately fixed in 4% paraformaldehyde for 24 hours, dehydrated, cleared, and embedded in paraffin. Paraffin sections 4 μm thick were prepared and stained with hematoxylin-eosin (HE) and immunohistochemistry for histological evaluation and protein-expression analysis.

### 3.8. Assessment of Cell Migration and Invasion

HepG2 and Huh7 hepatoma cells from the control or knockdown group were collected 48 hours after transfection and added to Transwell or invasion chambers at a density of 1 × 10^5^ cells per well, with 3 replicate wells per group. Next, 700 μL of DMEM containing 10% FBS was added to the lower chambers of 24-well plates, and the cells were incubated for 24 or 36 hours. Samples were stained with crystal violet for 30 minutes and then washed 3 times with PBS.

### 3.9. HUVEC Migration Assay

HepG2 and Huh7 hepatoma cells from the knockdown and control groups were harvested 48 hours after transfection, and conditioned medium was collected. Human umbilical vein endothelial cells (HUVECs) were added to Transwell chambers at a density of 10^5^ cells per well, with 3 replicate wells per group. The upper chambers were placed in 24-well plates containing 700 μL of culture medium in the lower chamber and incubated for 12 hours.

### 3.10. HUVEC Tube-Formation Assay

After 48 hours of transfection, HUVECs were seeded in 96-well plates coated with matrix gel. After 10 hours of incubation in conditioned medium (CM), calcein-AM fluorescence labeling was performed. Random fields of tubular structures were selected and imaged using an inverted fluorescence phase-contrast microscope for quantitative analysis.

### 3.11. Statistical Analysis

All quantitative data were analyzed using GraphPad Prism 9.0 and are presented as mean ± standard deviation (mean ± SD). For normally distributed data, as assessed by the Shapiro-Wilk test, with homogeneity of variance as assessed by the Levene test, independent-samples t tests were used to compare SERPINE1 expression levels in patient cancer tissues and adjacent tissues. Non-normally distributed data were analyzed using the Mann-Whitney U test. One-way analysis of variance (ANOVA) with Bonferroni post hoc correction was used for comparisons among multiple groups. Spearman rank correlation was used for correlation analysis, and survival was analyzed using Kaplan-Meier curves and log-rank tests. Statistical significance was set at a 2-tailed P < 0.05, with adjustment for multiple comparisons.

## 4. Results

### 4.1. Differential Expression and Prognostic Analysis of SERPINE1 in Liver Cancer

**Figure 1.**
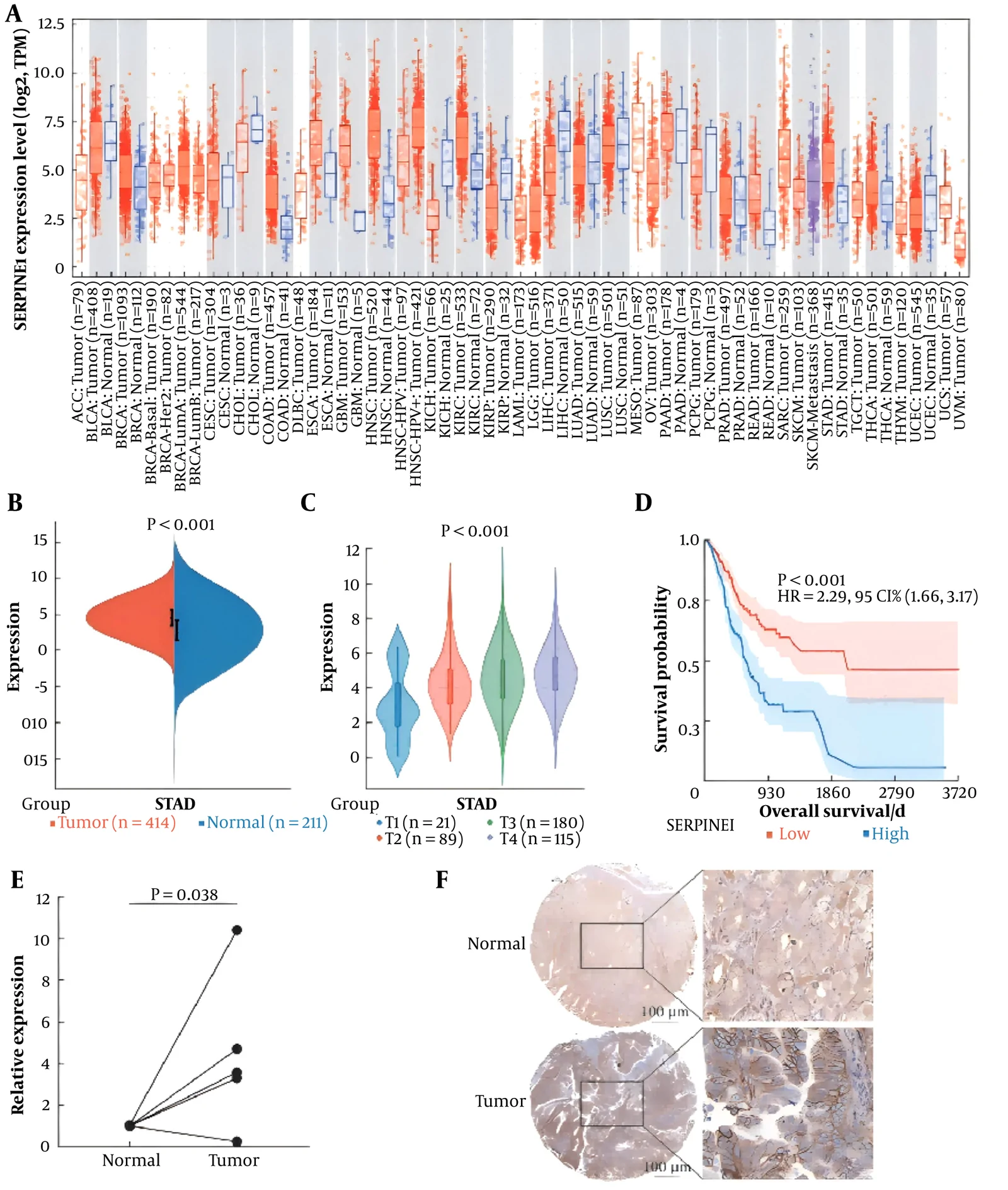
Differential expression of SERPINE1 in liver cancer. A. Pan-cancer analysis of SERPINE1 expression; B, TCGA database data on SERPINE1 expression in hepatocellular carcinoma; C, SERPINE1 expression at different stages of hepatocellular carcinoma; D, Survival curves for patients with different SERPINE1 expression levels; E, SERPINE1 expression in clinical hepatocellular carcinoma samples determined by qRT-PCR; F, SERPINE1 protein expression in normal and cancerous liver tissues determined by immunohistochemistry using data from the Human Protein Atlas (HPA) database (n = 100).

Pan-cancer analysis of SERPINE1 transcription in tumor and adjacent tissues was performed using the TIMER 2.0 online database (Figure 1A-C). Survival curves for patients with liver cancer were plotted according to SERPINE1 expression using clinical data. Overall survival was lower in the high-SERPINE1-expression group than in the low-expression group (P < 0.001) (Figure 1D). SERPINE1 was also significantly upregulated in liver cancer tissues (P = 0.038) (Figure 1E). Immunohistochemical images of normal and cancerous liver tissues were obtained from the Human Protein Atlas database (Figure 1F).

### 4.2. Effect of SERPINE1 Knockdown on Migration and Invasion of Hepatoma Cells

**Figure 2.**
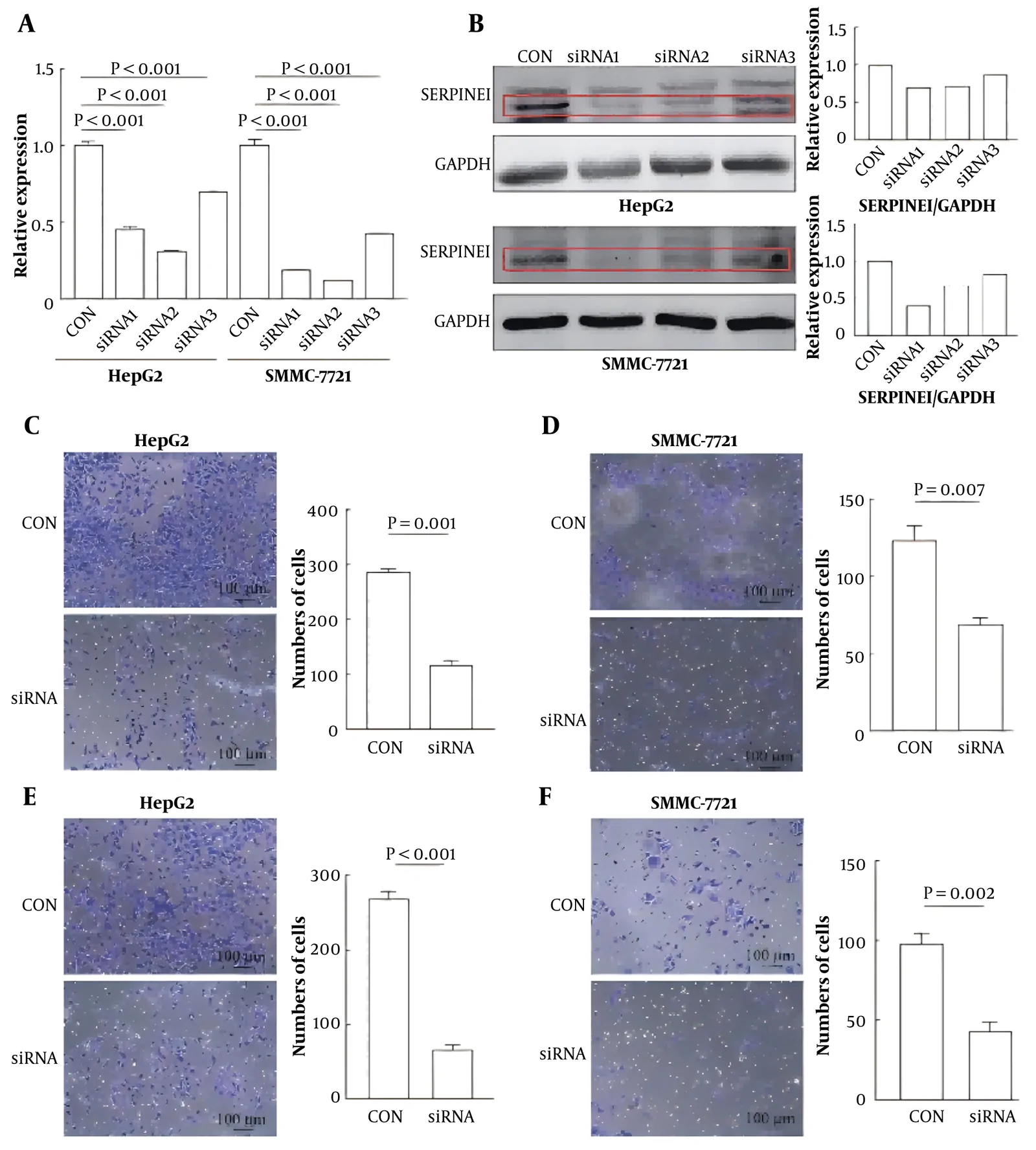
SERPINE1 knockdown inhibits migration and invasion in hepatocellular carcinoma. A-B. SERPINE1 interference efficiency in HepG2 and Huh7 cells was confirmed by qRT-PCR (A) and Western blotting (B). C-D. Transwell migration assays in HepG2 (C) and Huh7 (D) cells. E-F. Cell invasion was assessed using Transwell assays in HepG2 (E) and Huh7 (F) cells (n = 3).

The HCC cell lines HepG2 and Huh7 were used to investigate the effects of SERPINE1 knockdown via siRNA transfection. SERPINE1 mRNA and protein expression in the interference groups (siRNA1, siRNA2, and siRNA3) was markedly reduced in HepG2 and Huh7 cells (P < 0.05) (Figure 2A-B). Cell migration was evaluated using a Transwell assay (Figure 2C-D). A Matrigel-coated Transwell model was used to assess invasion and quantify invading cells in each group (Figure 2E-F).

### 4.3. Effects of SERPINE1 Knockdown on Angiogenesis in Hepatoma Cells

**Figure 3.**
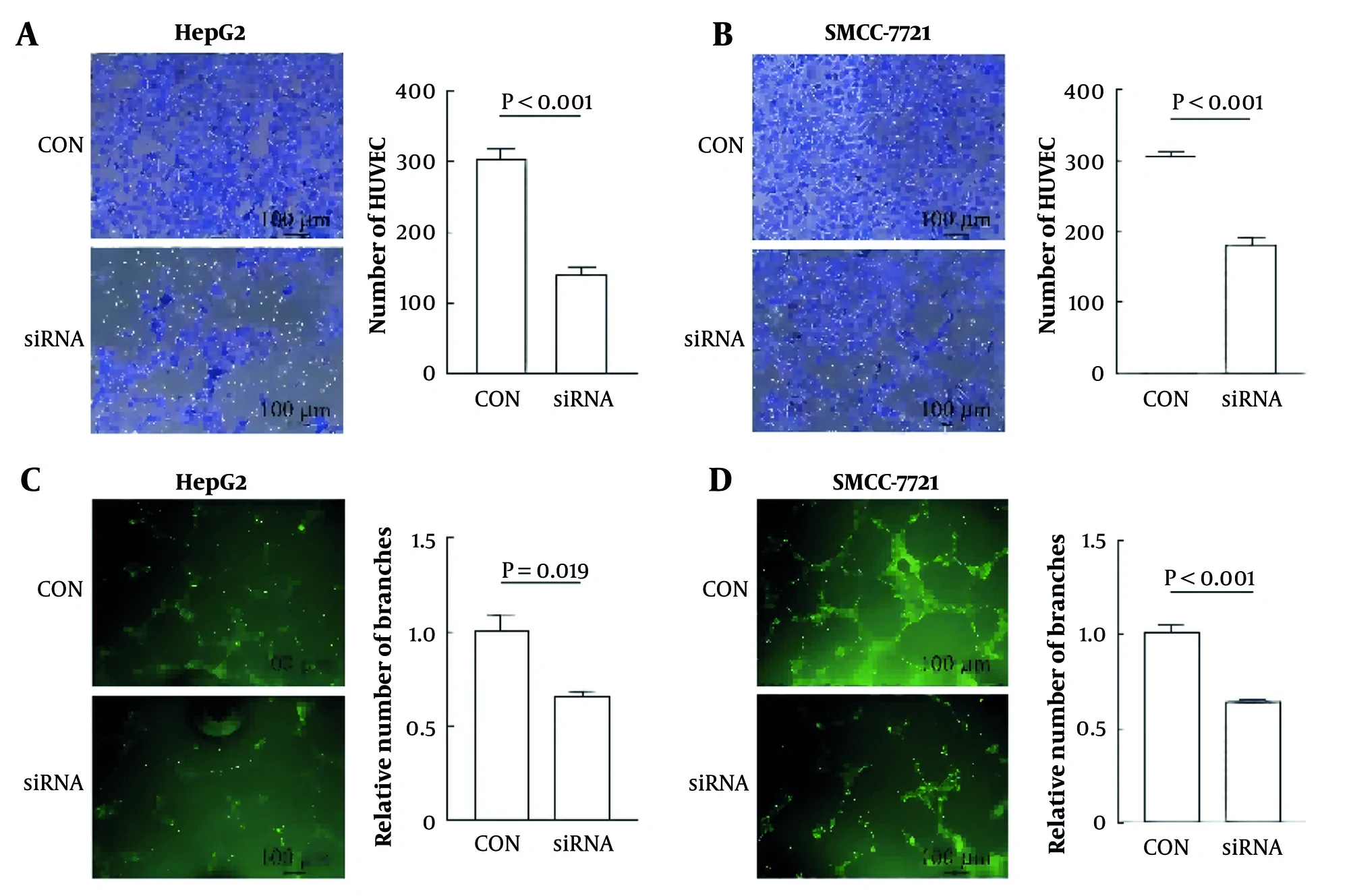
SERPINE1 knockdown inhibits angiogenesis in hepatocellular carcinoma. A-B. Transwell migration assays of HUVECs grown in conditioned medium from HepG2 (A) and Huh7 (B) cells. C-D. Tube formation of HUVECs grown in conditioned medium from HepG2 (C) and Huh7 (D) cells (n = 10).

The effect of SERPINE1 expression on angiogenesis in hepatoma cells was evaluated using HUVEC migration and tube-formation assays. In the HUVEC migration experiment (Figure 3A-B), SERPINE1 was specifically knocked down in HepG2 and Huh7 cells, and the culture supernatant was cocultured with HUVECs. The number of migrating HUVECs was significantly lower in the siRNA group (P < 0.001). Similar effects were evaluated in the HUVEC tube-formation experiment (Figure 3C-D).

### 4.4. Biological Functions and Enrichment Pathways Associated With SERPINE1

**Figure 4.**
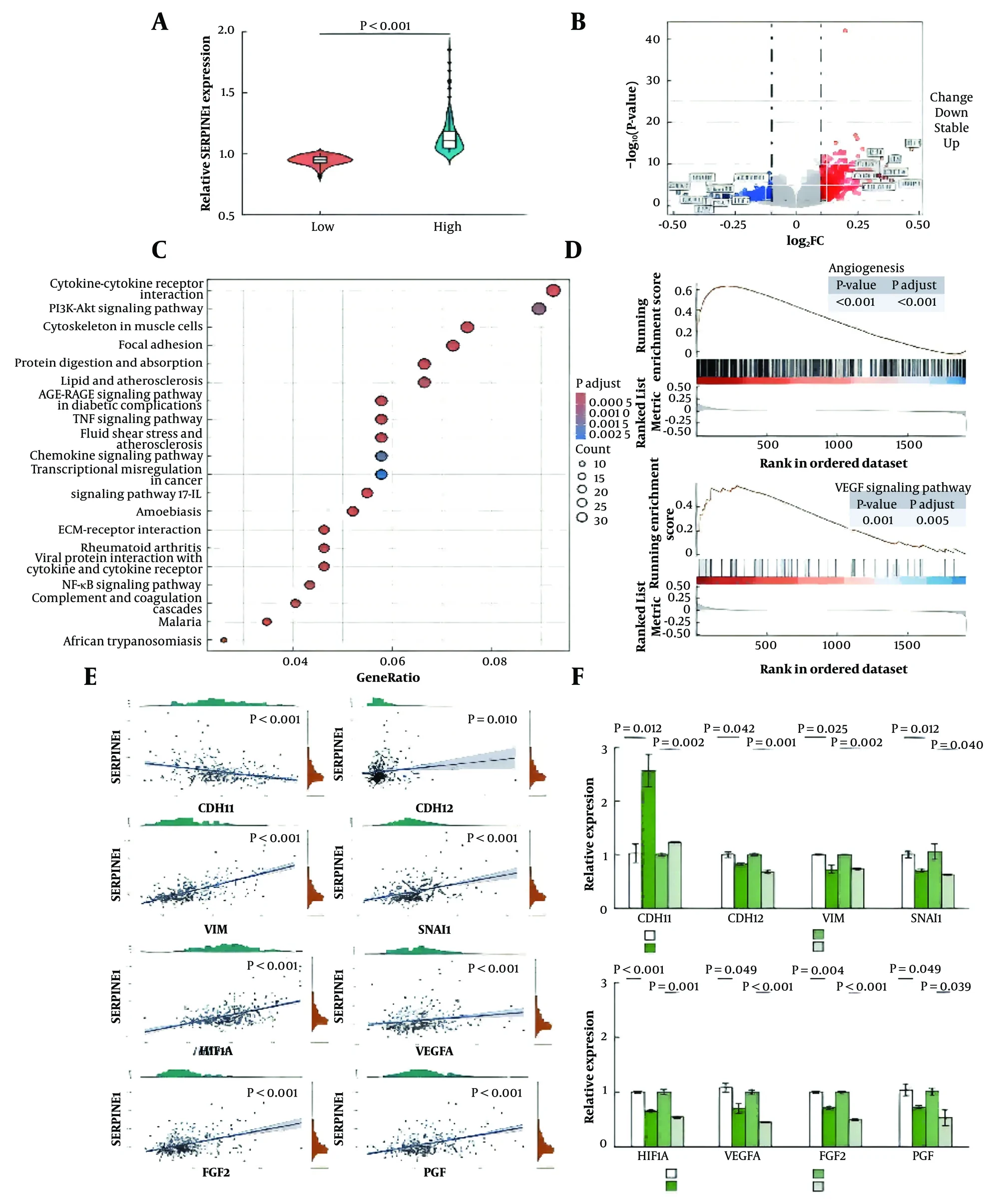
Validation of hepatocellular carcinoma mRNA levels and enrichment analysis of genes differentially expressed between SERPINE1 expression groups. A. SERPINE1 low- and high-expression groups; B, Differential gene-expression analysis of SERPINE1 high- and low-expression groups; C, KEGG enrichment analysis of SERPINE1 high- and low-expression groups; D, GSEA enrichment analysis of SERPINE1 high- and low-expression groups; E, Correlations between SERPINE1 expression and important genes involved in angiogenesis and EMT; F, qRT-PCR confirmation of genes associated with angiogenesis and EMT in HepG2 and Huh7 cells (n = 3).

The median SERPINE1 expression level was used to define groups for differential gene-expression analysis (logFC > 0.1, P < 0.05). A total of 200 downregulated genes and 475 upregulated genes were identified (Figure 4A-B). KEGG pathway analysis showed that the DEGs were significantly enriched in cancer-related pathways, including focal adhesion and extracellular matrix-receptor interactions (Figure 4C). SERPINE1 was associated with hepatoma-cell adhesion, migration, and invasion, and DEGs between SERPINE1 expression groups were further analyzed using gene set enrichment analysis (GSEA). Angiogenesis and the VEGF signaling pathway were positively correlated with SERPINE1 (Figure 4D), suggesting an association between SERPINE1 and angiogenesis in hepatic neoplasms. SERPINE1, a pivotal angiogenesis-related gene, was also associated with hypoxia-inducible factor 1α (Figure 4E).

## 5. Discussion

The early symptoms of liver cancer, a common malignant tumor worldwide, are often nonspecific, and regular screening rates are low (19-21). Current therapeutic efficacy in patients with highly heterogeneous liver cancer remains limited. To explore SERPINE1 expression in liver cancer and its potential molecular mechanisms (22), SERPINE1 was knocked down using siRNA. Further enrichment analysis using the GEO dataset indicated that high SERPINE1 expression could activate cancer-related pathways, including cell adhesion, extracellular matrix-receptor interaction, angiogenesis, and VEGF signaling (23-25). Epithelial-mesenchymal transition is the process by which epithelial cells acquire mesenchymal characteristics (26).

The results of multiple experiments provided preliminary evidence for a potential regulatory mechanism of SERPINE1 in the EMT process of HCC. Western blot analysis showed that SERPINE1 knockdown significantly inhibited TGF-β1-induced Smad2/3 phosphorylation, with p-Smad2/3 expression decreasing by ≥ 60%, and immunofluorescence tracing confirmed that SERPINE1 knockdown reduced the nuclear localization of the EMT transcription factors Snail and Slug. These findings suggest that SERPINE1 may participate in EMT regulation by enhancing TGF-β/Smad signaling. In the MAPK pathway, SERPINE1 overexpression significantly increased p-ERK1/2 and p-JNK levels, and the ERK inhibitor U0126 reversed its pro-invasive effect, with the number of cells in the Transwell assay decreasing by 52 ± 6% (P < 0.001), indicating that the MAPK pathway participates in SERPINE1-mediated cell invasion. The dual-luciferase reporter assay showed that SERPINE1 increased β-catenin/TCF4 transcriptional activity 2.8-fold, and this effect was blocked by the Wnt inhibitor ICG-001, suggesting that the Wnt/β-catenin pathway may participate in SERPINE1-mediated regulation of EMT-related genes such as vimentin.

This study provides only preliminary evidence regarding the interaction between SERPINE1 and these signaling pathways. The precise mechanism by which SERPINE1 regulates crosstalk among multiple signaling pathways requires further clarification. Future studies should define the role of SERPINE1 as a key molecule in the protein cascade of the tumor microenvironment and clarify differences in its regulation of the TGF-β, MAPK, and Wnt pathways under different pathological conditions.

Atypical reactivation of EMT is associated with malignant tumor-cell characteristics during progression and metastasis, including enhanced migration and invasion, increased tumor stemness, and resistance to chemotherapy and immunotherapy (27). Immunohistochemical analysis has suggested that high SERPINE1 expression may increase the risk of metastasis in head and neck cancer. SERPINE1 overexpression may further accelerate head and neck cancer-cell migration by activating the phosphatidylinositol 3-kinase/protein kinase B (PI3K/AKT) pathway. In HCC, hypoxia-inducible factor 2α (HIF-2α)-dependent induction of SERPINE1 promotes angiogenesis by reducing active hemagglutinin levels. Studies have shown that SERPINE1 can promote the proliferation, migration, and invasion of hepatoma cells, consistent with the findings of this study (28). However, this study focused on the effect of SERPINE1 on angiogenesis in liver cancer and suggested that SERPINE1 knockdown inhibits angiogenesis in liver cancer cells. Furthermore, bioinformatics analysis and qRT-PCR were used to preliminarily explore possible mechanisms by which SERPINE1 regulates hepatoma-cell migration, invasion, and angiogenesis. SERPINE1 may regulate tumor necrosis factor and thereby affect hepatoma-cell migration, invasion, and angiogenesis (29). This study has several limitations. For example, some data were obtained from public databases, preventing a complete assessment of their quality and accuracy.

This study focused on the core mechanism by which SERPINE1 promotes liver cancer invasion and metastasis through the EMT pathway. Recent studies suggest that SERPINE1 may participate in the regulation of the tumor immune microenvironment. Reports have shown that, in melanoma and lung cancer, SERPINE1 can induce macrophage polarization toward the M2 phenotype and promote the infiltration of regulatory T cells (Tregs), thereby creating an immunosuppressive microenvironment. Of particular interest is the potential association between SERPINE1 and immune-checkpoint molecules. In breast cancer models, activation of TGF-β signaling may indirectly promote PD-L1 expression, and TGF-β is also a central inducer of EMT. Transcriptional data from this study preliminarily indicate that after SERPINE1 silencing, the expression of interferon-response genes such as IRF1 and STAT1 is significantly upregulated in liver cancer cells. These genes are typically positively correlated with antigen presentation and T-cell activation, suggesting that SERPINE1 may weaken the antitumor immune response by inhibiting interferon signaling. TCGA database association analysis showed a positive trend between high SERPINE1 expression in liver cancer tissues and PD-L1 (CD274) mRNA levels (P = 0.052), although the association did not reach statistical significance (P > 0.05).

The immune-related evidence provided by this study remains preliminary: 1) PD-L1 protein expression levels were not measured; 2) the distribution of immune cells in the tumor microenvironment was not directly observed; and 3) the causal relationship between SERPINE1 and PD-L1 was not verified by functional experiments. Future studies should use multiplex immunofluorescence to systematically analyze the effects of SERPINE1 deletion on the distribution of PD-L1-positive cells and lymphocyte infiltration in the liver cancer microenvironment and verify its regulatory effects on immune-cell function using in vitro coculture models, thereby providing stronger experimental evidence for evaluating SERPINE1 as a potential target for enhancing immunotherapy.

### 5.1. Conclusions

Bioinformatics analysis and phenotypic experiments evaluating liver cancer-cell migration, invasion, and angiogenesis showed that SERPINE1 is upregulated in liver cancer tissues and may contribute to disease progression. This study revealed high SERPINE1 expression in liver cancer and a potential molecular regulatory mechanism, providing a new perspective for understanding liver cancer invasion and metastasis. Although these results suggest that SERPINE1 may be a potential target for liver cancer research, its clinical value for prognostic assessment and treatment requires further verification in larger clinical studies and functional experiments. Future research should focus on the specific mechanism of SERPINE1 in the liver cancer microenvironment and the effects of interventions targeting this molecule on tumor progression, thereby providing a theoretical basis for precision treatment of liver cancer.

## Data Availability

The dataset presented in the study is available on request from the corresponding author during submission or after publication.
